# Follow the sign! Top-down contingent attentional capture of masked arrow cues

**DOI:** 10.2478/v10053-008-0091-3

**Published:** 2011-12-01

**Authors:** Heiko Reuss, Carsten Pohl, Andrea Kiesel, Wilfried Kunde

**Affiliations:** Department of Psychology, Julius-Maximilians-Universität Würzburg, Germany

**Keywords:** attention, arrow cues, spatial cuing, masked priming, contingent capture

## Abstract

Arrow cues and other overlearned spatial symbols automatically orient attention according to their spatial meaning. This renders them similar to exogenous cues that occur at stimulus location. Exogenous cues trigger shifts of attention even when they are presented subliminally. Here, we investigate to what extent the mechanisms underlying the orienting of attention by exogenous cues and by arrow cues are comparable by analyzing the effects of visible and masked arrow cues on attention. In Experiment 1, we presented arrow cues with overall 50% validity. Visible cues, but not masked cues, lead to shifts of attention. In Experiment 2, the arrow cues had an overall validity of 80%. Now both visible and masked arrows lead to shifts of attention. This is in line with findings that subliminal exogenous cues capture attention only in a top-down contingent manner, that is, when the cues fit the observer’s intentions.

## Introduction

Our ability to focus cognitive resources on behaviorally relevant stimuli enables us to efficiently act and interact with our environment. This selection process is, amongst other things, achieved through spatial shifts of attention. These shifts of attention can happen in two ways, which both have been investigated extensively (e.g., Folk, Remington, & Johnston, 1992; Jonides, 1981; Müller & Rabbitt, 1989; Posner, 1980; Posner & Cohen, 1984; Theeuwes, 1991; Yantis & Johnson, 1990). On the one hand, shifts of attention can be initiated intentionally by the observer, for example, because a task like a visual search task demands shifting attention to several locations in the visual field to find a target (Treisman & Gelade, 1980; Treisman, Sykes, & Gelade, 1977; Wolfe, 1994), or because we follow a sign or a cue stimulus that informs us about the likely location of a target stimulus (Posner, 1980; Posner, Snyder, & Davidson, 1980). This kind of shift of attention is often referred to as being endogenous, and is thus thought to reflect an intentional orienting of attention under internal cognitive control. On the other hand, sudden stimulus onsets, like a loud bang, or a flash of light, automatically draw our attention to them, without our intention to do so (e.g., Jonides & Yantis, 1988). This automatic capture of attention is called exogenous, which refers to the external aspect of this kind of orienting of attention.

Endogenous and exogenous shifts of attention have distinctive confining features. These features were investigated, for example, with the spatial cuing paradigm (Posner, 1980; Posner et al., 1980) in which a cue informs a participant about the location of the following target. Thereby, cues are either valid (i.e., the cue correctly informs a participant about the actual location of the target) or invalid (the cue signals a location where the target does not appear). The difference in response time (RT) between trials with invalid cues and trials with valid cues (the validity effect) is an indicator for shifts of attention, as this difference results from facilitated processing of the target stimulus (because its location is already attended to) after valid cues as compared to the necessity to first reorient attention to the target location after invalid cues (Posner, 1980).

Validity effects occur even if the cue is not related to a certain response (because a participant has to respond to the identity of the target, not to its location, which is cued). Thus, the validity effect cannot be attributed to response priming. Instead it is assumed that participants orient their attention to the cued location. This assumption is further supported by electrophysiological measurements that provide evidence for the link between validity effects and orienting of attention (e.g., Ansorge & Heumann, 2006; Vossel, Thiel, & Fink, 2006).

In the spatial cuing paradigm, the nature of the cue, as well as the nature of its influence on our attentional system, can be varied. First, there are exogenous cues, which are sudden stimulus onsets, typically a change in luminance, that occur directly at the possible target location. Exogenous cues lead to shifts of attention even when the cues indicate the actual location of the target only at chance level so that there is no overall benefit for a participant to attend to the cued location (e.g., Folk & Remington, 1998, 1999; Posner, 1980; Remington, Folk, & McLean, 2001). Even when participants are instructed to ignore the cues, the cues capture attention (Jonides, 1981). This reflects the automatic and externally driven nature of exogenous orienting of attention.

Second, there are endogenous cues that are presented centrally, for example, letters or signs that indicate one of the possible target locations. For these cues, the mapping of cue to location is arbitrary. Thus, the cues have to be interpreted first, before the participant can then shift attention according to the cues’ meaning. This shift of attention is endogenously initiated by a participant and there usually needs to be a benefit for the participants to shift attention according to the instruction. In contrast to exogenous cues, endogenous cues have to predict the target location above chance level (e.g., valid cues in 80% of trials) in order to lead to shifts of attention.

 There are cases, however, in which centrally presented cues lead to shifts of attention even if they do not predict the target location above chance level. Some symbolic cues seem to have an automatic effect akin to that of exogenous cues. First, it was found that social cues like pointing gestures (Langton, Watt, & Bruce, 2000) or eye gaze (Driver et al., 1999; Friesen & Kingstone, 1998; Kunde, Skirde, & Weigelt, 2011; Langton & Bruce, 1999) automatically trigger orienting of attention. If, for example, eyes that gaze either to the left or to the right (two possible target locations) are centrally presented as cues, attention is oriented to the cued location even if the eye gaze cues correctly predict the target location only in 50% of the trials. Second, the same was found for symbolic cues that have a very strong spatial meaning, like the words right or left, or arrows pointing in one direction (Eimer, 1997; Friesen, Ristic, & Kingstone, 2004; Gibson & Bryant, 2005; Hommel, Pratt, Colzato, & Godijn, 2001; Pratt, Radulescu, Guo, & Hommel, 2010; Tipples, 2002). For example, Eimer (1997) showed both with behavioral and with electrophysiological data that centrally presented arrow cues impact on attention even when they are not informative. It seems that seeing a conventional, overlearned symbol with a spatial meaning automatically directs one’s visual attention to the location this symbol designates (Hommel et al., 2001). Consequently, it was argued that eye gaze and arrow cues trigger shifts of attention that are truly reflexive, and not under volitional control (Stevens, West, Al-Aidroos, Weger, & Pratt, 2008). In a way, these symbolic cues automatically influence attention similarly to exogenous cues. 

 Exogenous cues that are masked still have the power to capture attention (e.g., Ansorge & Heumann, 2006; Ansorge & Neumann, 2005; Ivanoff & Klein, 2003; Lambert, Naikar, McLachlan, & Aitken, 1999; McCormick, 1997; Mulckhuyse, Talsma, & Theeuwes, 2007; Scharlau, 2002; Scharlau & Ansorge, 2003; Scharlau & Neumann, 2003; for a review, see Mulckhuyse & Theeuwes, 2010). During masking, the visibility of a brief visual stimulus is reduced, sometimes down to a level of invisibility so that a participant is not aware of the stimulus, by subsequent or preceding visual masking stimuli near or at the location of the masked stimuli (in this case, the cue). In one of the first studies on this subject, which also illustrates the difference between endogenous and exogenous orienting of attention, McCormick (1997) used peripheral bars as cues that participants were either aware or unaware of. Participants were instructed not to attend to the location where the cue appeared, but to the opposite location, where the target would appear in 85% of the trials. McCormick reasoned that the cue would first capture attention exogenously, but when participants were aware of the cue, they would reorient their attention endogenously away from the cue as instructed. This should result in faster RTs when the target appears at the opposite location than when the target appears at the same location as the cue. When participants were unaware of the cue, however, no endogenous reorienting should occur, and enhanced performance when the target appears at the location of the cue would demonstrate that the cues captured attention exogenously. The results indeed indicated that subliminal cues captured attention, as RTs were shorter when the target appeared at the location of the cue than when it appeared at the other location (one should note, however, that an alternative account based on inhibition of return [Klein, 2000] is also viable to explain this pattern of results). This also shows that participants were not able to reorient their attention endogenously according to the instruction when they were unaware of the cue, which underlines the strong connection between awareness and endogenous control (Posner & Snyder, 1975). When participants were aware of the cue, they reoriented their attention and, thus, RTs were shorter when the target appeared at the likely location opposite of the cue than at the unlikely location. 

 In elaborating determinants of subliminal exogenous cuing, Ansorge and Neumann (2005) investigated if masked singleton-cues are able to draw attention to them, and further if this attentional capture is purely stimulus-driven or depends on top-down settings, that is, active intentions of the participant. First, their results showed that masked cues were able to trigger shifts of attention. Participants responded faster after valid than after invalid cues, even when the cues were masked. Second, they found that attentional capture only worked when the cues’ features were fitting those of the task settings. For example, in Experiment 2, participants had to respond to red bars, but the cues were black, not red. The effect of the masked cues was virtually eliminated. The authors concluded that the effect of masked exogenous cues depends on the participant’s intention, as governed by the task instructions. If masked cues do not match control settings which are set up according to the instruction to search for a target with certain features, the cues have no or only a very minor effect. More recently, it has been shown that only task-relevant features of subliminally presented color singletons captured spatial attention, while cues that did not match top-down settings (i.e., with a task-irrelevant color) did not (Ansorge, Horstmann, & Worschech, 2010; see also Held, Ansorge, & Müller, 2010). 

 Thus, masked exogenous cuing effects seem to be restricted to top-down-contingent capture. This means that a cue captures attention only when it fits to current top-down settings of the participant (e.g., when it fits to current search templates). Folk et al. (1992) demonstrated this phenomenon for unmasked cues. They showed that cues that appeared at possible target locations captured attention only when the cues shared the feature used to locate the target (e.g., abrupt onset or specific color). Folk and colleagues concluded that attentional control settings are a function of behavioral goals. Events or stimuli that exhibit goal-corresponding features are able to capture attention. Such attentional capture is thus dependent on top-down-settings, and not per se dependent on overall cue validity. As we will argue later, however, overall cue validity can influence top-down settings and thus modulate contingent capture. 

More evidence that masked singleton-cues are able to capture attention, but that this effect is contingent on top-down control settings, comes from perceptual latency priming (Scharlau, 2002; Scharlau & Ansorge, 2003; Scharlau & Neumann, 2003). When a masked cue precedes a visible target, the target is perceived as temporally leading another comparison stimulus. This is interpreted as facilitated processing of the target due to attentional capture of the cue. This effect, however, is absent or reduced when the cue does not have a target-like shape or color (Scharlau & Ansorge, 2003).

The rationale behind our study was that overlearned spatial cues (like arrows) are able to orient attention automatically in a way that seems similar to that of exogenous cues. Thus, masked arrow cues might be able to orient attention in the same way as masked exogenous cues do. Furthermore, we tested if such influences on attention depend on top-down settings. We assumed that when cues are informative regarding the target’s location, participants would more likely have the intention to use the cues to guide their attention than when cues are not informative with regard to the target’s location. To this end, we conducted two experiments that used the spatial cuing paradigm. In both experiments, participants were presented either a visible or a masked central arrow cue and then had to respond to a peripheral target. We varied the overall cue validity such that in Experiment 1 cues were not predictive, that is, overall cue validity was 50% (with two target locations), and participants had no incentive to prepare to use the cues. In Experiment 2, the cues were predictive with an overall cue validity of 80%, so that participants were likely to intentionally use the arrow cues and to set up fitting top-down settings as this would benefit their performance.

## Experiment 1

### Method

#### Participants

Twenty students (11 female, 9 male) of the University of Würzburg with an average age of 25 years participated in individual sessions of approximately 50 min either in fulfillment of course requirements or in return for payment (6 €). All participants reported normal or corrected-to-normal vision, and were naive with respect to the hypothesis of the experiment.

#### Apparatus and Stimuli

An IBM compatible computer with a 17 inch VGA-Display and the software package E-Prime™ (Schneider, Eschman, & Zuccolotto, 2002) were used for stimulus presentation and response sampling. Stimulus presentation was synchronized with the vertical retraces of a 100-Hz monitor, resulting in a refresh rate of 10 ms. Viewing distance was approximately 60 cm. Responses were executed with the index fingers of both hands and collected with external response keys. All stimuli were presented in black on a white background.

A central arrow extending 2.5 × 1.0 cm was used as cue, pointing either to the right or left side. The arrow was either metacontrast-masked (see Breitmeyer, 1984) by a larger rectangle extending 3.9 × 2.3 cm with an inner edge fitting exactly the contours of the arrow cues, or non-masked by just being underlined with a horizontal residual line of the mask extending 2.5 × 0.3 cm. The letters *X* or *O*, typed in bold Courier New font with a font size of 24 pixels, served as targets and were presented either on the right or on the left side, with a distance of 11.3 cm to the center of the screen.

#### Procedure and Design

**Figure 1.**
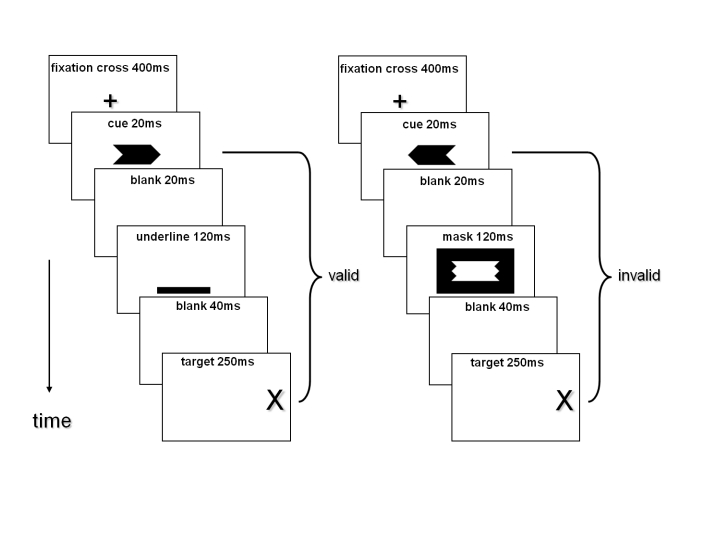
Sequence of stimuli in trials in Experiments 1 and 2. On the left, the sequence of stimuli in a trial with a non-masked arrow cue that is valid is illustrated. On the right, the sequence of stimuli in a trial with a masked arrow cue that is invalid is illustrated.

The sequence of events in a trial is depicted in Figure 1. Each trial started with a central fixation cross extending 0.7 x 0.7 cm that was presented for 400 ms. Following the fixation cross, a central arrow cue was presented for two refresh cycles of the display (20 ms). After cue presentation a blank white screen was displayed for 20 ms followed either by a metacontrast mask or an underline that was presented for 120 ms, followed again by a blank white screen, displayed for 40 ms. At last, the target letter was presented for 250 ms. Participants had to respond within a time window of 5,000 ms after target onset. After response execution a fixed time interval of 1,000 ms elapsed before the next trial started. All eight different possible combinations of cue (left/right-pointing arrow), masked/non-masked, target identity (*X/O*), and target side (left/right) were repeated 80 times each (for a total of 640 trials altogether), and conditions varied randomly on a trial-by-trial-basis. Thus, the arrow cue had an overall validity of 50%. After every 64 trials, participants were allowed a short, self-paced break.

Participants were informed that an arrow was to appear before target onset in some of the trials, and that the arrow was pointing correctly to the side where the target letter was to appear in 50% of these trials. They were instructed to look first at the fixation cross and then to respond as fast and as accurately as possible to the identity of the laterally presented target letter. Half of the participants had to press a left key when the letter *O* was presented and a right key when the letter *X* was presented. For the other half of the participants, the mapping was reversed. Errors were indicated by the German word for *wrong* (“Falsch!”) presented in red in the lower part of the monitor. Response times were recorded from the onset of the target stimulus until a response was given.

#### Assessment of Cue Visibility

Avisibility test with 128 trials with non-masked and masked arrow cues was applied directly after the main experiment. Participants were fully informed about the structure of a trial and the presence of masked (and non-masked) cues. They had to perform a forced-choice discri-mination task. For this task, the sequence of stimuli was exactly the same as in the main experiment. However, there was no time limit after target onset. Participants were asked to discriminate whether a right- or a left-pointing arrow was presented, and had to press either “1” (for a left-pointing arrow) or “0” (for a right-pointing arrow) on the number pad of the keyboard. Participants could freely choose which fingers to use for this task. Participants were instructed to take their time, and to try to be as accurate as possible. They were also encouraged to guess if they thought they had not seen anything, and were reminded to bear in mind that the probability for a left- or right-pointing arrow was equal. In order to avoid that unconscious priming effects influence the free response choice (Kiesel, Wagener, et al., 2006; Schlaghecken & Eimer, 2004), response keys in the cue visibility task differed as compared to the main experiment and, additionally, there was an interval of 750 ms after target offset in which no response was possible (see Vorberg, Mattler, Heinecke, Schmidt, & Schwarzbach, 2003).

### Results

#### Validity effects

**Figure 2.**
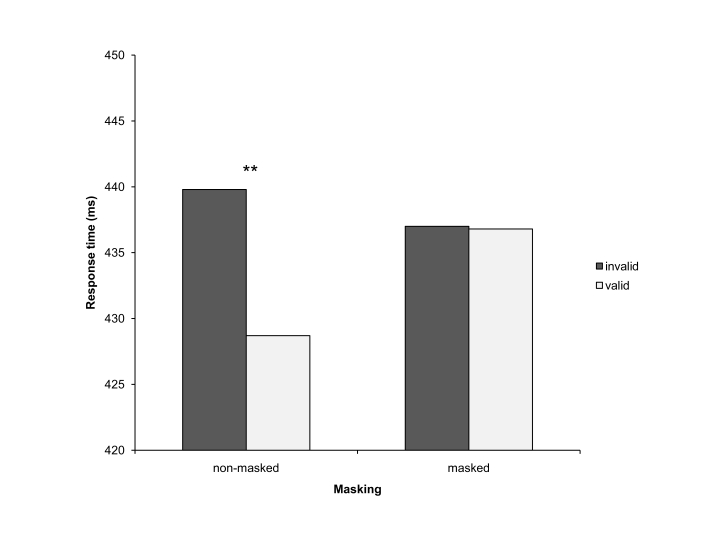
Response times (RTs) in Experiment 1 (overall cue validity 50%). The dark grey bars represent RTs in trials with invalid cues, the light grey bars represent RTs in trials with valid cues. The bars in the left column depict RTs with non-masked cues, and the bars in the right column depict RTs with masked cues. ** indicates effects with *p*< .01.

Trials with RTs deviating more than 2.5 standard deviations from the mean RT of each participant and each condition were excluded (2.1%). Mean RTs for correct responses were submitted to the analysis of variance (ANOVA) with the within-subject factors Masking (masked and non-masked cue) and Validity (valid and invalid cue). The factor Validity was significant, *F*(1, 19) = 10.2, *p*< .01, η^2^ = .35, as well as the interaction of Masking × Validity, *F*(1, 19) = 7.0, *p*< .05, η^2^ = .27. The main effect of Masking was not significant, *p*= .16. Single comparisons revealed that only non-masked cues, *t*(19) = 3.4, *p*< .01, but not masked cues, *t*(19) = 0.8, *p*= .93, elicited a validity effect. Participants responded 11 ms faster to valid than to invalid non-masked arrow cues, while for masked cues there was no difference (0 ms) in RT between responses to invalid and to valid cues (see Figure 2).

The overall mean error rate was 7.9%. The same ANOVA of error rates revealed no significant effects (*ps* ≥ .26).

#### Cue Visibility

Cue visibility was assessed by computing the signal detection mea-sure d’, separately for non-masked and masked arrow cues, treating the right-pointing arrow cue as signal and the left-pointing arrow cue as noise. Hits and false alarms proportions were corrected according to the log-linear rule if participants had 0% hits or 100% false alarms (Goodman, 1970, as cited in Hautus, 1995).

Participants’ discrimination performance for the non-masked cues was d’ = 3.18, with a mean hit rate of 90.7 % and a mean false alarm rate of 9.6 %, and deviated from zero, *t*(19) = 12.09, *p*< .001. Participants’ discrimination performance for the masked cues was *d*’ = 0.64, with a mean hit rate of 70.0 % (i.e., when a right pointing arrow was present), and a mean false alarm rate of 49.2 %, and deviated from zero, *t*(19) = 3.68, *p*< .01. Thus, there was a response bias to indicate a right-pointing cue in trials with masked cues, as this response was given in 59.6% of these trials. The d’ values for non-masked and masked arrow cues differed significantly from each other, *t*(38) = 8.58, *p*< .001.

### Discussion

In Experiment 1, we found that centrally presented arrow cues lead to shifts of attention although they are not predictive of the target location. With non-masked cues, participants responded 11 ms faster after valid cues than after invalid cues. This validity effect reflects shifts of attention following the arrow cue, which result in facilitated target processing after valid cues and hampered target processing after invalid cues because of the necessity to reorient attention to the target location. This result replicates the finding that endogenous cues with a strong spatial meaning, like arrows, impact on attention akin to exogenous cues (Hommel et al., 2001; Pratt et al., 2010; Tipples, 2002).

With masked cues, however, no validity effect was found. In contrast to masked exogenous cues, masked arrow cues did not induce shifts of attention. Considering that an “overlearned symbol with a spatial meaning automatically directs one’s visual attention” (Hommel et al., 2001, p. 364), it seems rather counterintuitive that this automatic effect depends on the conscious experience of the arrow and cannot operate outside of awareness. However, it was repeatedly shown that the effects of masked exogenous cues are not purely bottom-up and stimulus-driven, but that attentional capture strongly depends on top-down control settings (e.g., Ansorge & Heumann, 2006; Ansorge & Neumann, 2005; Ivanoff & Klein, 2003; Scharlau, 2002; Scharlau & Ansorge, 2003; Scharlau & Neumann, 2003). With the experimental design of Experiment 1, participants had no incentive to orient their attention according to the cue. The cues were not predictive regarding the actual location of the target, and participants were not explicitly instructed to orient their attention according to the cue. Therefore, we conjecture that participants did not form the top-down setting, or “action plan”, to use the arrows by shifting attention to the indicated location. With visible arrow cues, the impact of the overlearned stimulus is so strong that an effect occurs exogenously without such an action plan. Masked cues, however, presumably only impact the system if it is set up accordingly.

To test this assumption, we ran Experiment 2 with an overall cue validity of 80%. With this manipulation, the arrow cues were predictive regarding the target’s location, and participants most likely formed an action plan to use the arrow cues to shift their attention.

## Experiment 2

### Method

#### Participants

Twenty students who had not participated in Experiment 1 participated in individual sessions of approximately 50 min either in fulfillment of course requirements or in return for payment (6 €). Participants were 17 females and three males with an average age of 22 years. All participants reported normal or corrected-to-normal vision, and were naive with respect to the hypothesis of the experiment.

#### Apparatus, Stimuli, Procedure, and Design

Apparatus, stimuli, procedure, and design were identical to Experiment 1, except for the following changes: The arrow cues had a validity of 80%, so that the target appeared in the cued location in 80% of the trials, and in the other location in 20% of the trials. All other combinations of cue (left/right-pointing arrow), presence of a mask or a line, target identity (*X/O*), and target side (left/right) varied orthogonally, with the target side corresponding to the arrow in 80% of the trials. In total, there were 40 trials, of which 32 were valid and eight were invalid trials, in a block, which was run 18 times (720 trials altogether). After every 120 trials, participants were allowed a short, self-paced break. Participants were informed that the non-masked arrow is pointing to 80% correctly to the location where the target letter will appear.

#### Assessment of Cue Visibility

Assessment of cue visibility was identical to Experiment 1, except for the fact that in Experiment 2, 192 trials with non-masked and masked arrow cues were applied as visibility test directly after the main experiment. In contrast to the main experiment, the arrow cues were no longer predictive to the side where the target letter would appear, as otherwise participants would be able to construe from the target’s location which arrow was probably shown. Participants were informed about this.

### Results

#### Validity effects

**Figure 3.**
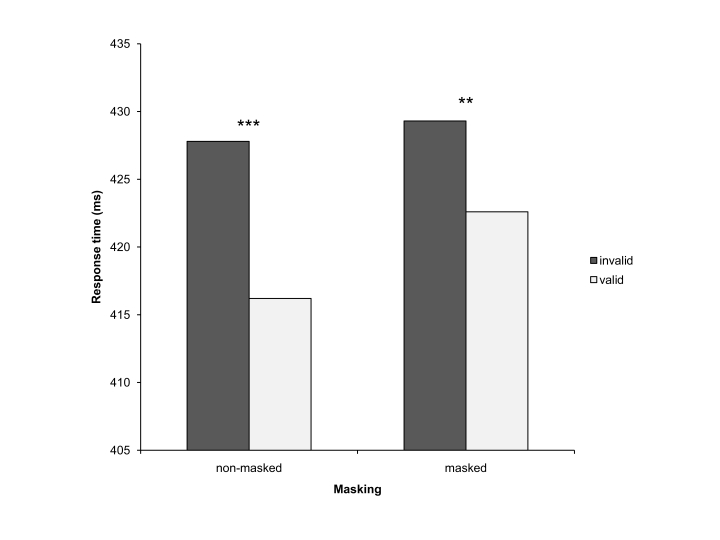
Response times (RTs) in Experiment 2 (overall cue validity 80%). The dark grey bars represent RTs in trials with invalid cues, the light grey bars represent RTs in trials with valid cues. The bars in the left column depict RTs with non-masked cues, and the bars in the right column depict RTs with masked cues. ** indicates effects with *p*< .01. *** indicates effects with *p*< .001.

Trials with RTs deviating more than 2.5 standard deviations from the mean RT of each participant and each condition were excluded (2.1 %). Mean RTs for correct responses were submitted to the analysis of variance (ANOVA) with the within-subject factors Masking (masked and non-masked cue) and Validity (valid and invalid cue). The factor Masking was significant, *F*(1, 19) = 5.1, *p*< .05, η^2^ = .21, as well as the factor Validity, *F*(1, 19) = 19.4, *p*< .001, η^2^ = .51. The interaction Masking × Validity was not significant, *F*(1, 19) = 2.4, *p*= .13. Participants responded faster to non-masked (422 ms) than to masked (426 ms) arrow cues. Single comparisons revealed a validity effect for non-masked cues, *t*(19) = 3.9, *p*< .001, as well as for masked cues, *t*(19) = 3.1, *p*< .01. Participants responded 12 ms faster to valid than to invalid non-masked arrow cues, and 7 ms faster to valid than to invalid masked arrow cues (see Figure 3).

The overall mean error rate was 7.0%. The same ANOVA of error rates revealed no significant effects at all (*ps* ≥ .64).

To formally compare the results of both experiments, an additional ANOVA with the within-participant factor Validity (valid and invalid) and the between-participants factor Experiment (Experiment 1 with 50 % overall cue validity and Experiment 2 with 80 % overall cue validity) was conducted for RTs in both experiments for masked arrow cues only. The factor Validity was significant, *F*(1, 38) = 5.3, *p*< .05, as well as the interaction Validity × Experiment, *F*(1, 38) = 4.8, *p*< .05. This interaction reflects that the validity of the arrow cue influenced RTs in Experiment 2, while no such effect was present in Experiment 1.

#### Cue Visibility

Cue visibility was analyzed as in Experiment 1. Participants’ discrimination performance for the non-masked cues was d’ = 3.45, with a mean hit rate of 91.7 % and a mean false alarm rate of 8.8 %, and deviated from zero, *t*(19) = 11.53, *p*< .001. Participants’ discrimination performance for the masked cues was *d*’ = 0.69, with a mean hit rate of 73.5 % and a mean false alarm rate of 51.3 %, and deviated from zero, *t*(19) = 3.18, *p*< .01. These data again show a response bias to indicate a right-pointing cue in trials with masked cues, as this response was given in 62.4% of the trials. Again, as in Experiment 1, the d’ values for non-masked and masked arrow cues differed significantly from each other, *t*(38) = 7.44, *p*< .001. Comparing the results for the visibility of the cues between Experiments 1 and 2, neither the d’ values for non-masked cues, *t*(38) = -0.41, *p*= .68, nor for masked cues, *t*(38) = -0.181, *p*= .86, differed significantly.

### Discussion

In Experiment 2, participants responded faster after valid than after invalid arrow cues. In contrast to Experiment 1, this validity effect was present not only for visible, but also for masked arrow cues, and indicates that shifts of attention were triggered both by visible and masked cues. The critical difference to Experiment 1 was that overall cue validity was raised from 50% to 80%. With cues that are predictive of the target’s location above chance level, participants are likely to form intentions to use the cues, as this would benefit their performance. This intention seems to be crucial for masked cues to have an effect on attention.

When comparing the validity effects of Experiments 1 and 2, it seems surprising that the validity effect with visible cues did not increase in Experiment 2, but was virtually the same as in Experiment 1. One might expect a larger validity effect with the additional incentive to use the cues provided by their increased validity. One reason why this was not the case might be the relatively short cue-target SOA (stimulus onset asynchrony) used in the experiments at hand (200 ms). We conjecture that endogenously driven shifts of attention emerged too slowly to be observable in the RT data. So on the one hand, the intention to use the cues enabled masked cues to automatically trigger shifts of attention that occurred rapidly and thus were observable in a validity effect. On the other hand, possible endogenously initiated shifts of attention that are due to this intention emerged too late after cue onset so that they could not effectively influence RTs and thus did not add to the size of the validity effect. As our experiments only had one fixed SOA, this hypothesis is of course speculative and would need to be corroborated (or dismissed) by similar experiments that implement different and especially longer SOAs.

## General Discussion

We conducted two experiments to investigate the effect of visible and masked arrow cues on attention. We were able to replicate findings that visible, centrally presented arrows trigger automatic shifts of attention (Friesen et al., 2004; Gibson & Bryant, 2005; Hommel et al., 2001; Pratt et al., 2010; Tipples, 2002). Most importantly, masked arrow cues also triggered shifts of attention, yet only when overall cue validity was 80%, whereas masked cues remained ineffective when overall cue validity was 50%. Thus, our results showed that with masked arrows, the effect of centrally presented arrows is not purely stimulus driven, but modulated by the partcipants’ current intentions and top-down settings.

In recent studies, arrow cues, among other stimuli, like eye gaze cues (Driver et al., 1999; Friesen & Kingstone, 1998; Langton & Bruce, 1999), have been found to have automatic effects on attention when presented centrally as spatial cues. Usually, centrally presented spatial cues only affect attention if the observer intends to shift attention according to the cue. We conjecture that the observer endogenously controls these shifts of attention, and they are only initiated if cue validity is above chance level so that the cues benefit performance. Arrow cues, however, seem to have a more automatic effect on attention. Presumably due to their overlearned spatial meaning, attention is automatically oriented towards the indicated location by arrows, regardless of cue validity.

Such automatic capture of attention can otherwise be observed with exogenous cues that appear directly at target location. Exogenous cues even have the power to orient attention towards them when they are presented subliminally, which underlines the automatic nature of the effect (Ansorge & Heumann, 2006; Ansorge & Neumann, 2005; Ivanoff & Klein, 2003; Lambert et al., 1999; McCormick, 1997; Mulckhuyse et al., 2007). It was also found, however, that this exogenous attentional capture is not a solely stimulus-driven effect, but is contingent on the cues matching the participant’s top-down settings. The first aim of our experiments was therefore to test if masked arrow cues affect attention. Our second aim was to investigate whether possible effects of masked arrow cues on attention are purely stimulus-driven or depend on top-down settings.

In both experiments, participants had to respond to a laterally presented target by pressing one of two response keys. In each trial, an arrow cue appeared in the center of the screen. In half of the trials, however, the arrow was metacontrast-masked by a following stimulus. In Experiment 1, the arrows had an overall validity of 50% and therefore were not predictive of the target’s location. With visible arrows, we found a validity effect. Participants responded faster when the arrow pointed to the location of the target than when the target appeared on the other side. This reflects shifts of attention to the indicated location and thereby facilitated processing when the target actually appeared there, but hindered processing when attention had to be reoriented first when the target appeared on the other side. This result replicated earlier works on the effect of spatial symbols like arrows on the orienting of attention (Hommel et al., 2001; Pratt et al., 2010; Tipples, 2002).

 When the arrow was masked, however, RTs were not influenced by the validity of the arrow, and thus attention was not shifted according to the arrow’s direction. This result is in line with studies that observed that masked exogenous cues have to fit to the top-down settings, that is, the cues’ features have to fit to the current intentions and action plans to be able to capture attention (Ansorge & Heumann, 2006; Ansorge, Heumann, & Scharlau, 2002; Ansorge, Kiss, & Eimer, 2009; Ansorge & Neumann, 2005). For example, when participants in a study by Ansorge and Neumann (2005) had to respond to red stimuli, exogenous cues that were black did not draw attention while exogenous cues that were red did. 

Alternatively, it is possible that participants tried to actively ignore the cues because the participants knew the cues had no actual value in helping to locate the target. Maybe participants were successfully ignoring masked arrows. Non-masked arrows, however, still impacted on attention and thus it seems they could not be ignored successfully. This again would parallel the effects of exogenous peripheral cues that also capture attention if participants were instructed to ignore the cues (Jonides, 1981).

To investigate whether top-down settings are crucial for the effects of masked arrow cues on attention, in Experiment 2 overall cue validity was raised to 80% to encourage participants to use the arrow cues, and to form fitting intentions and top-down settings. With visible arrow cues, we again found a validity effect that reflected shifts of attention to the location indicated by the cue. Importantly, a validity effect was also present with masked arrow cues. Thus, in contrast to Experiment 1, attention was oriented according to the masked arrows.

As the visibility tests of both experiments did not result in different measures of sensitivity between experiments (with d’ values of 0.64 and 0.69), this result cannot be attributed to differences in the visibility of the masked cues, but is due to the manipulation of cue validity and the corresponding top-down settings. In Experiment 1, participants had no incentive and, thus, most likely no intention to orient their attention according to the arrows (or possibly even tried to actively ignore the cues). Clearly visible arrows still had an impact on attention because of their overlearned spatial meaning, but when participants were presumably not aware of the arrow, the missing top-down settings to orient attention accordingly when an arrow is perceived was crucial and prevented the masked cues from having an effect. In Experiment 2, the incentive and the intention to use the cues was provided by the high cue validity. This top-down control setting enabled the masked arrows to impact on attention.

Remarkably, previous research that investigated whether subliminally presented central arrow stimuli impact on behavior, that is, motor responses, also comes to the conclusion that top-down settings are crucial for masked arrows to exert an effect (Eimer & Schlaghecken, 1998; Klapp, 2009; Klapp & Haas, 2005; Klapp & Hinkley, 2002; Schlaghecken & Eimer, 2004; Schlaghecken, Klapp, & Maylor, 2009). This assumption is also in line with many studies on masked priming effects indicating that subliminally presented stimuli generally impacted on behavior only if the prime fits the current top-down setting/current intentions (e.g., Ansorge, 2006; Kiefer, 2007; Kiefer & Brendel, 2006; Kiesel, 2009; Kiesel, Kunde, & Hoffmann, 2007; Kiesel, Kunde, Pohl, & Hoffmann, 2006; Kunde, Kiesel, & Hoffmann, 2003, 2005; Martens & Kiefer, 2009; Pohl, Kiesel, Kunde, & Hoffmann, 2010). Taken together, these results show that the effects of masked stimuli both on behavior and on attention are based on strikingly similar preconditions. Further research might investigate if these similarities are due to the oftentimes proposed close link between attention and the motor system (e.g., Rizzolatti, Riggio, Dascola, & Umiltá, 1987), or if there is a basic mechanism of unconscious processing that also applies to the priming of other processes besides motor or attentional processes.

To conclude, we assume that the underlying mechanisms of how masked arrows induce spatial shifts of attention are comparable to the mechanisms of how masked exogenous cues trigger shifts of attention. Both have effects on attention that seem automatic in nature. These effects, however, are not purely stimulus-driven, but depend on current top-down settings.
